# Supplementary material for: Strain-dependent contribution of the AcrAB-TolC efflux pump to Klebsiella pneumoniae physiology
Source: Microbiology (Reading). 2025 Dec 19;171(12):001647. doi: 10.1099/mic.0.001647 (PMC12715972; doi:10.1099/mic.0.001647)
Supplement: Uncited Supplementary Material 1. [file mic-171-01647-s001.pdf]

Supplementary Material

Strain-dependent contribution of the AcrAB-TolC efflux pump to *Klebsiella pneumoniae* physiology

Kirandeep Bhogal[1,2], Barbara Clough[1,3], Charlotte Emmerson[1,2], Archie Organ[1,2], Yin Chen[1,3], Michelle MC Buckner[1,2], Ilyas Alav[1,2,4#]

[1] Institute of Microbiology and Infection, University of Birmingham, Birmingham, UK

[2] Department of Microbes, Infection and Microbiomes, School of Infection, Inflammation and Immunology, College of Medicine and Health, University of Birmingham, Birmingham, UK

[3] School of Biosciences, College of Life and Environmental Sciences, University of Birmingham, Birmingham, UK

[4] Present address: Sir William Dunn School of Pathology, University of Oxford, United Kingdom, UK

[#] Corresponding author

Email: ilyas.alav@path.ox.ac.uk

Supplementary Table 1. List of primers used for generating the *acrB* and *ramR* mutant *Klebsiella pneumoniae* Ecl8 and ATCC 43816 strains.

Primer description	Primer sequence (5'-3')
Forward primer for amplifying the kanamycin resistance gene from pKD4 with flanking sites that have homology to the *acrB* gene in *Klebsiella pneumoniae* Ecl8 and ATCC 43816	CGCGCCATCAGAACAAACCAAGTCTTAACTTAAACAGGAGCCGTTAAGACG TGTAGGCTGGAGCTGCTTC
Reverse primer for amplifying the kanamycin resistance gene from pKD4 with flanking sites that have homology to the *acrB* gene in *Klebsiella pneumoniae* Ecl8 and ATCC 43816	CCAGTGATAAAAAAGGGCCGCGGTAGCGGCCCTTTGTTCAGGAGTGAAG AGGGAATTAGCCATGGTCCAT
Forward primer that binds upstream of the *acrB* gene in *Klebsiella pneumoniae* Ecl8 and ATCC 43816	TACCGCAACAGGGTGTTACC

Reverse primer that binds downstream of the *acrB* gene in *Klebsiella pneumoniae* Ecl8 and ATCC 43816	CAGCTGTCGCGTTCAGAAAC
Forward primer for amplifying the kanamycin resistance gene from pKD4 with flanking sites that have homology to the *ramR* gene in *Klebsiella pneumoniae* Ecl8 and ATCC 43816	CAAGATCGGCGGTTTGTTTAAACCTGCGTGAGGAAAAAAGTAGTGTAGGCTGGAGCTGCTTC
Reverse primer for amplifying the kanamycin resistance gene from pKD4 with flanking sites that have homology to the *ramR* gene in *Klebsiella pneumoniae* ATCC 43816	TCAGGGCGATACGGTGAGCGCAGGGATGCAGCATCTCAGGGGGGGAATTAGCCATGGTCCAT
Reverse primer for amplifying the kanamycin resistance gene from pKD4 with flanking sites that have homology to the *ramR* gene in *Klebsiella pneumoniae* Ecl8	AGGGCGATACGGTGAGCGCAGGGATGCAGTGTTTCCGGCGTCAGGGAATTAGCCATGGTCCAT

Forward primer that binds upstream of the *ramR* gene in *Klebsiella pneumoniae* Ecl8 and ATCC 43816	ACTGGTTATGGAACTGGCCG
Reverse primer that binds downstream of the *ramR* gene in *Klebsiella pneumoniae* Ecl8 and ATCC 43816	CCCGCGAATAGTCATGGTGA

Supplementary Table 2. List of primers used for the real-time PCR reactions.

Gene	Forward (Fwd) and reverse (Rev) primer sequences (5'-3')	Amplicon size (bp)	Primer efficiency	R^2
rpoB	Fwd: CACCCTTGTTACCGTGACGA<br>Rev: AAATCACCCAAGGCGACGAT	120	102%	0.999
acrA	Fwd: GGTGCCCAACAGTTTCTGAT<br>Rev: GTCCTCAGGTCAGTGGCATT	200	99.3%	0.992
acrB	Fwd: CCGCATGTTTGATAAGAGCAC<br>Rev: GATACAGCACCAGATAACGACC	86	98.2%	0.997
tolC	Fwd: TAGTGTTGCCGTAGCCATC<br>Rev: GTAAACCCATCTCCACCTCC	84	102.6%	0.997
ramA	Fwd: ATCGTCGAGTGGATTGATGA<br>Rev: AGATGCCATTTCGAATACCC	86	98.5%	0.987
ramR	Fwd: GCGCAATATCTGGAACAGCTA<br>Rev: CGGAAAGCTCTCTTTTACCTG	130	104%	0.957

All primers had an annealing temperature of 60 °C

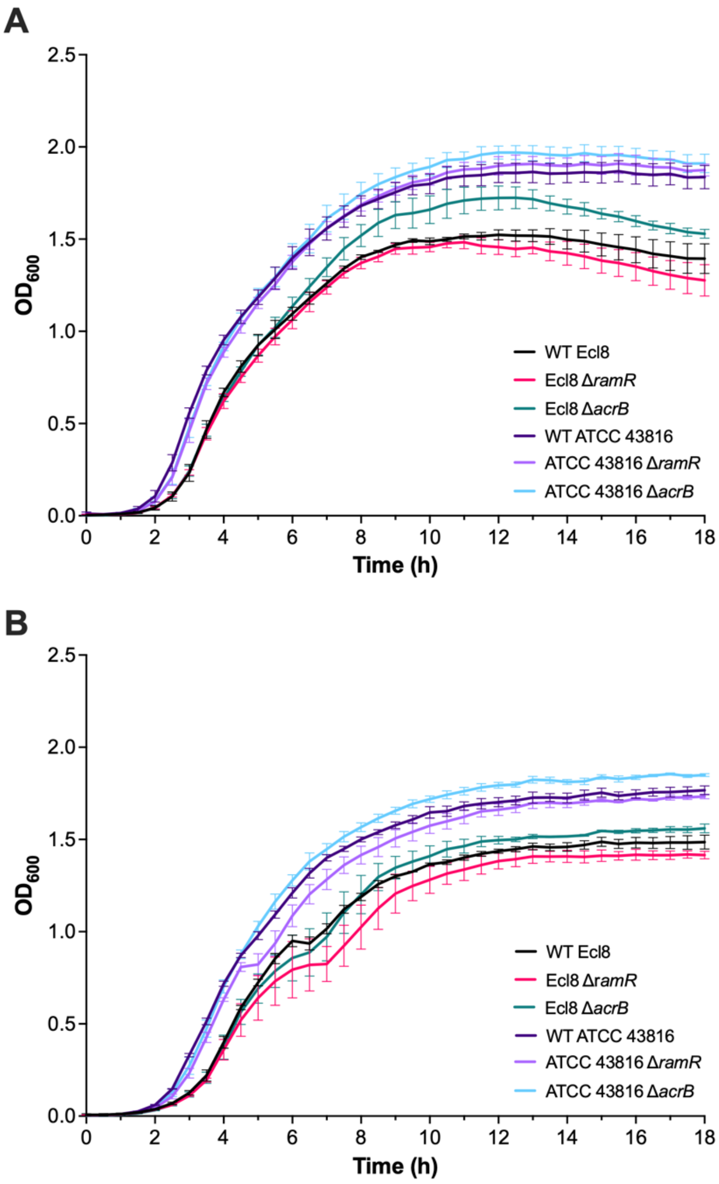

Figure S1. The growth of wild-type *Klebsiella pneumoniae* Ecl8 and ATCC 43816, as well as their isogenic Δ*ramR* and Δ*acrB* mutant strains, in Luria-Bertani (LB) or cation-adjusted Mueller-Hinton broth (CAMHB). The growth kinetics of bacterial cells were measured in **A)** LB broth or **B)** CAMHB**.** The optical density at 600 nm (OD_{600}) was measured every 30 min over 18 h at 37 °C with shaking (200 rpm) using a plate reader. The data presented are the mean ± standard deviation of three independent experiments, each consisting of three biological replicates.

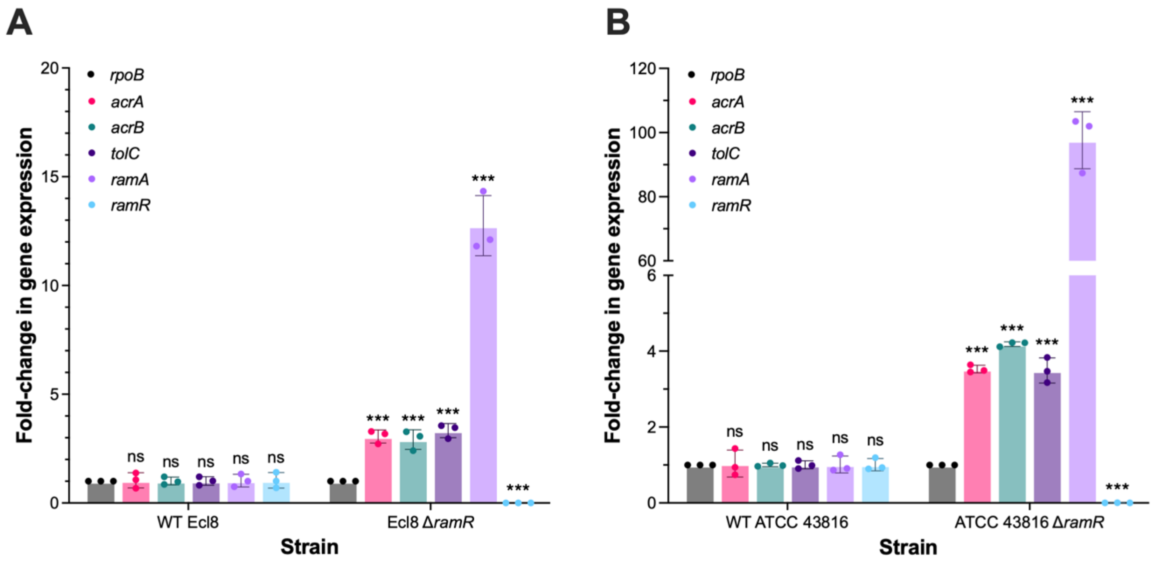

Figure S2. The effect of *ramR* deletion on *acrA*, *acrB*, *tolC* and *ramA* expression in *Klebsiella pneumoniae* Ecl8 and ATCC 43816. Relative fold change in gene expression of *acrA*, *acrB*, *tolC*, *ramA,* and *ramR* in **A)** wild-type *K. pneumoniae* Ecl8 and its isogenic Δ*ramR* strain and **B)** wild-type *K. pneumoniae* ATCC 43816 and its isogenic Δ*ramR* strain. The relative expression of *acrA*, *acrB*, *tolC*, *ramA,* and *ramR* was determined in bacteria grown in LB broth to the exponential phase (OD_{600} = 0.5) compared to the *rpoB* gene. The data presented are the mean ± standard deviations from three biological replicates. For each strain, statistical significance was determined by comparing the relative expression of *rpoB* to the genes of interest using one-way ANOVA, followed by Dunnett's test to correct for multiple comparisons. Significantly different results are presented and are indicated with *** ($P \leq 0.001$). ns, not significant.

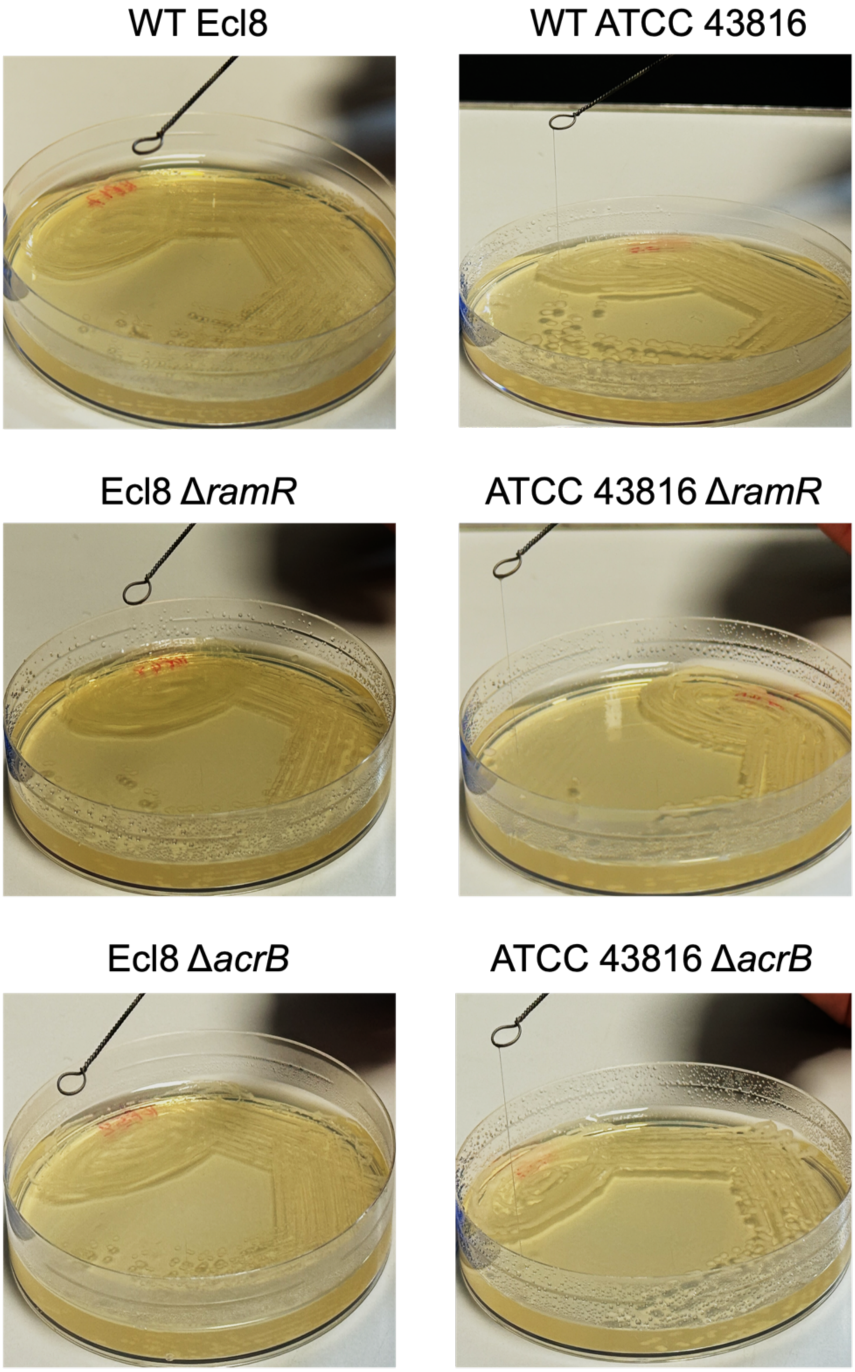

Figure S3. String test for *Klebsiella pneumoniae* Ecl8 and ATCC 43816 strains. *K. pneumoniae* Ecl8 and its isogenic Δ*ramR* and Δ*acrB* mutant strains do not exhibit a hypermucoviscous phenotype. *K. pneumoniae* ATCC 43816 and its isogenic Δ*ramR* and Δ*acrB* mutant strains exhibit a hypermucoviscous phenotype, indicated by the formation of a string from the stretching of colonies.
